# Correction to “HPV vaccination following cervical intraepithelial neoplasia grade 2 diagnosis and risk of progression”Eriksen DO, Krog L, Ostenfeld EB, et al. HPV vaccination following cervical intraepithelial neoplasia grade 2 diagnosis and risk of progression. Acta Obstet Gynecol Scand. 2026;105(2):377–385. doi:10.1111/aogs.70128.

**DOI:** 10.1111/aogs.70199

**Published:** 2026-04-08

**Authors:** Dina Overgaard Eriksen

**Affiliations:** ^1^ University Clinic for Gynecological HPV‐Related Disease, Department of Obstetrics and Gynecology Gødstrup Hospital Herning Denmark; ^2^ Department of Clinical Medicine Aarhus University Aarhus Denmark; ^3^ NIDO Centre for Research and Education Gødstrup Hospital Herning Denmark

In the originally published version of this article, the hazard ratio and adjusted hazard ratio estimates reported in Table 2 and Tables S1 and S2 contained minor numerical inaccuracies due to a specification error in the analysis code. The estimates have been corrected in the text below and the updated tables are provided. The revised hazard ratios are slightly lower in magnitude but remain consistent in direction. Confidence intervals, statistical inference, and the interpretation of the findings are overall unchanged, and the conclusion of the study remains unaffected.

**TABLE 2:** Risk of CIN3+ among women managed by active surveillance for CIN2 by HPV vaccination status, overall and by age, year of diagnosis, and index cytology.

	HPV vaccination status	Total, *n*	CIN3+, *n*	CIF, % (95% CI) 5 years	HR (95% CI)	aHR^c^ (95% CI)
Overall	Total CIN2	4585	1391	29.9 (28.5–31.3)		
	Vaccinated	583	201	33.7 (29.8–37.6)	1.15 (0.99–1.33)	1.19 (1.02–1.38)
	Unvaccinated	4002	1190	29.3 (27.9–30.8)	Ref. 1.00	Ref. 1.00
Age at CIN2 diagnosis						
18–29	Vaccinated	434	151	33.7 (29.2–38.2)	1.21 (1.02–1.45)	1.24 (1.04–1.48)
	Unvaccinated	2292	662	27.9 (26.0–29.8)	Ref. 1.00	Ref. 1.00
30–40	Vaccinated	149	50	33.1 (25.4–40.9)	1.09 (0.81–1.46)	1.14 (0.85–1.53)
	Unvaccinated	1710	528	31.2 (28.9–33.5)	Ref. 1.00	Ref. 1.00
Year of CIN2 diagnosis						
2007–2012	Vaccinated	440	146	31.6 (27.2–36.0)	1.16 (0.97–1.38)	1.16 (0.97–1.39)
	Unvaccinated	2277	677	27.3 (25.5–29.2)	Ref. 1.00	Ref. 1.00
2013–2020	Vaccinated	143	55	40.2 (31.7–48.5)	1.31 (0.99–1.72)	1.34 (1.01–1.78)
	Unvaccinated	1725	513	31.9 (29.5–34.3)	Ref. 1.00	Ref. 1.00
Index cytology						
Nonhigh‐grade^a^	Vaccinated	311	86	26.7 (21.8–31.9)	1.09 (0.87–1.37)	1.13 (0.89–1.42)
	Unvaccinated	2182	538	23.9 (22.1–25.8)	Ref. 1.00	Ref. 1.00
High grade^b^	Vaccinated	272	115	41.6 (35.6–47.5)	1.20 (0.98–1.46)	1.31 (1.07–1.60)
	Unvaccinated	1820	652	35.8 (33.6–38.1)	Ref. 1.00	Ref. 1.00

^a^ Atypical squamous cells of undetermined significance or low‐grade squamous intraepithelial lesion and missing/other.

^b^ High‐grade squamous intraepithelial lesion, atypical glandular cells, atypical squamous cells cannot exclude HSIL, adenocarcinoma in situ, or carcinoma.

^c^ Adjusted for age at CIN2 diagnosis, calendar year, index cytology result (high‐grade vs. nonhigh‐grade), income level (lowest, middle, highest), and educational level (low, intermediate, high).

In the Abstract, the corrected Results are:

We included 4585 women, of whom 583 (12.7%) were vaccinated within 6 months after CIN2 diagnosis. A total of 1391 (30.3%) progressed to CIN3+ during follow‐up. The 5‐year cumulative risk was 29.9% (28.5–31.3). Overall, no protective effect of vaccination after CIN2 diagnosis was found (aHR 1.19 [1.02–1.38]). Stratified analyses showed increased progression risk with vaccination among women <30 years, in the latest calendar period (2013–2020), and among women with high‐grade index cytology; no significant difference in risk was observed in women ≥30 years, women with nonhigh‐grade index cytology, or in the early calendar period (2007–2013).

The second, third, and fourth paragraphs in the corrected Results section are:

Among the 4585 women included, 1391 (30.3%) progressed to CIN3+ during follow‐up. Cervical cancer was diagnosed in 18 women (0.4%). The 5‐year cumulative risk of CIN3+ was slightly higher among vaccinated women (33.7% [95% CI: 29.8–37.6]) compared to unvaccinated women (29.3% [95% CI: 27.9–30.8]) (Table 2, Figure 2). After adjusting for confounders, HPV vaccination was associated with an increased risk of progression to CIN3+, with an adjusted hazard ratio (aHR) of 1.19 (95% CI: 1.02–1.38) compared to unvaccinated women. When stratifying by age at the time of CIN2 diagnosis, we found that HPV vaccination was associated with an increased risk of progression among women <30 years (aHR 1.24 [95% CI: 1.04–1.48]), whereas there was no difference between vaccinated and unvaccinated women when aged ≥30 years (aHR 1.14 [95% CI: 0.85–1.53]). When stratifying by calendar period, vaccinated women had an aHR of 1.16 (95% CI: 0.97–1.39) compared with unvaccinated women among those diagnosed between 2007 and 2012, whereas in the most recent period (2013–2020), the risk of progression to CIN3+ was slightly higher among vaccinated compared with unvaccinated women (aHR 1.34 [95% CI: 1.01–1.78]). When stratifying by index cytology, vaccinated women had an aHR of 1.13 [95% CI: 0.89–1.42] compared with unvaccinated women among women with nonhigh‐grade cytology and an aHR of 1.31 (95% CI: 1.07–1.60) among those with high‐grade cytology at diagnosis (Table 2).

Only 14 women (0.3%) were diagnosed with vulva HSIL+, VaIN2+, or AIN2+ after their CIN2 diagnosis, precluding us from calculating cumulative risk and HRs.

In the first sensitivity analysis, where the exposure window was extended to 6 months before to 6 months after CIN2 diagnosis, estimates were slightly higher than in the main analysis, but it did not change the main conclusion (Table S1). When restricting the analysis to women vaccinated before their first follow‐up visit, the overall estimate was similar (Table S2). Adjusting for maternal income and educational level instead of the women's own socioeconomic variables did not change overall findings (data not shown).

We regret the error.

## Supporting information


**Table S1.** Risk of CIN3+ by HPV vaccination status overall and by age, year of diagnosis, and index cytology for women vaccinated 6 months before to 6 months after CIN2 diagnosis.
**Table S2.** Risk of CIN3+ by HPV vaccination status overall and by age, year of diagnosis, and index cytology for women vaccinated before their first follow‐up visit.

