# Supplementary material for: Correction to “HPV vaccination following cervical intraepithelial neoplasia grade 2 diagnosis and risk of progression”Eriksen DO, Krog L, Ostenfeld EB, et al. HPV vaccination following cervical intraepithelial neoplasia grade 2 diagnosis and risk of progression. Acta Obstet Gynecol Scand. 2026;105(2):377–385. doi:10.1111/aogs.70128
Source: Acta Obstet Gynecol Scand. 2026 Apr 8;105(6):1177–8. doi: 10.1111/aogs.70199 (PMC13191808; doi:10.1111/aogs.70199)
Supplement: Supplementary file 1 — Table S1. Risk of CIN3+ by HPV vaccination status overall and by age, year of diagnosis, and index cytology for women vaccinated 6 months before to 6 months after CIN2 diagnosis. Table S2. Risk of CIN3+ by HPV vaccination status overall and by age, year of diagnosis, and index cytology for women vaccinated before their first follow‐up visit. [file AOGS-105-1177-s001.docx]

**Supplementary Table 1**. Risk of CIN3+ by HPV vaccination status overall and by age, year of diagnosis, and index cytology for women vaccinated six months before to six months after CIN2 diagnosis

|  | HPV vaccination status | Total, n | CIN3+, n | CIF (%)  (95% CI)  5 years | HR  (95% CI) | aHR^c^  (95% CI) |
| --- | --- | --- | --- | --- | --- | --- |
| Overall | Total CIN2 | 4,911 | 1,503 | 30.1 (28.8-31.2) |  |  |
|  | Vaccinated | 909 | 313 | 33.5 (30.4-36.6) | 1.16 (1.02-1.31) | 1.20 (1.06-1.36) |
|  | Unvaccinated | 4,002 | 1,190 | 29.3 (27.9-30.8) | Ref. 1.00 | Ref. 1.00 |
| **Age at CIN2 diagnosis** | | | | | | |
| 18-29 | Vaccinated | 726 | 254 | 33.8 (30.3-37.3) | 1.24 (1.07-1.43) | 1.24 (1.07-1.43) |
|  | Unvaccinated | 2,292 | 662 | 27.9 (26.0-29.8) | Ref. 1.00 | Ref. 1.00 |
| 30-40 | Vaccinated | 183 | 59 | 31.9 (25.1-39.0) | 1.04 (0.79-1.36) | 1.09 (0.83-1.43) |
|  | Unvaccinated | 1,710 | 528 | 31.2 (28.9-33.5) | Ref. 1.00 | Ref. 1.00 |
| **Year of CIN2 diagnosis** | | | | | | |
| 2007-2012 | Vaccinated | 587 | 194 | 31.5 (27.7-35.3) | 1.16 (0.98-1.36) | 1.16 (0.99-1.37) |
|  | Unvaccinated | 2,277 | 677 | 27.3 (25.5-29.2) | Ref. 1.00 | Ref. 1.00 |
| 2013-2020 | Vaccinated | 322 | 119 | 36.9 (31.5-42.3) | 1.21 (0.99-1.47) | 1.28 (1.04-1.56) |
|  | Unvaccinated | 1,725 | 513 | 31.9 (29.5-34.3) | Ref. 1.00 | Ref. 1.00 |
| **Index cytology** | | | | | | |
| Non-high-grade^a^ | Vaccinated | 485 | 131 | 26.0 (22.1-30.1) | 1.06 (0.88-1.29) | 1.09 (0.90-1.32) |
|  | Unvaccinated | 2,182 | 538 | 23.9 (22.1-25.8) | Ref. 1.00 | Ref. 1.00 |
| High-grade^b^ | Vaccinated | 424 | 182 | 41.9 (37.1-46.6) | 1.23 (1.05-1.45) | 1.30 (1.10-1.54) |
|  | Unvaccinated | 1,820 | 652 | 35.8 (33.6-38.1) | Ref. 1.00 | Ref. 1.00 |

^a^Atypical squamous cells of undetermined significance or low-grade squamous intraepithelial lesion and missing/other

^b^ High-grade squamous intraepithelial lesion, atypical glandular cells, atypical squamous cells cannot exclude HSIL, adenocarcinoma in situ, or carcinoma.

^c^Adjusted for age at CIN2 diagnosis, calendar year, index cytology result (high-grade vs. non high-grade), income level (lowest, middle, highest) and educational level (low, intermediate, high).

**Supplementary Table 2**. Risk of CIN3+ by HPV vaccination status overall and by age, year of diagnosis, and index cytology for women vaccinated before their first follow-up visit

|  | HPV vaccination status | Total, n | CIN3+, n | CIF (%)  (95% CI)  5 years | HR  (95% CI) | aHR^d^  (95% CI) |
| --- | --- | --- | --- | --- | --- | --- |
| Overall | Total CIN2 | 5,308 | 2,114 | 26.8 (25.5-28.2) |  |  |
|  | Vaccinated | 554 | 254 | 32.5 (28.1-37.0) | 1.26 (1.06-1.50) | 1.29 (1.09-1.54) |
|  | Unvaccinated | 4,754 | 1,860 | 26.2 (24.8-27.6) | Ref. 1.00 | Ref. 1.00 |
| **Age at CIN2 diagnosis** | | | | | | |
| 18-29 | Vaccinated | 406 | 185 | 32.4 (27.3-37.5) | 1.27 (1.04-1.56) | 1.24 (1.01-1.52) |
|  | Unvaccinated | 2,711 | 1,019 | 25.3 (23.5-27.2) | Ref. 1.00 | Ref. 1.00 |
| 30-40 | Vaccinated | <150^c^ | 69 | 32.1 (23.8-40.8) | 1.32 (0.96-1.82) | 1.41 (1.02-1.95) |
|  | Unvaccinated | >2,040^c^ | 841 | 27.3 (25.1-29.6) | Ref. 1.00 | Ref. 1.00 |
| **Year of CIN2 diagnosis** | | | | | | |
| 2007-2012 | Vaccinated | 402 | 183 | 32.3 (27.3-37.4) | 1.19 (0.97-1.45) | 1.22 (1.00-1.50) |
|  | Unvaccinated | 2,795 | 1,120 | 27.4 (25.6-29.2) | Ref. 1.00 | Ref. 1.00 |
| 2013-2020 | Vaccinated | 152 | 71 | 32.7 (24.0-41.7) | 1.43 (1.02-2.02) | 1.50 (1.06-2.11) |
|  | Unvaccinated | 1,959 | 740 | 24.1 (21.8-26.5) | Ref. 1.00 | Ref. 1.00 |
| **Index cytology** | | | | | | |
| Non-high-grade^a^ | Vaccinated | 288 | 104 | 26.9 (21.5-32.6) | 1.22 (0.95-1.57) | 1.21 (0.94-1.56) |
|  | Unvaccinated | 2,497 | 812 | 21.5 (19.7-23.3) | Ref. 1.00 | Ref. 1.00 |
| High-grade^b^ | Vaccinated | 266 | 150 | 39.7 (32.8-46.6) | 1.32 (1.05-1.67) | 1.39 (1.10-1.77) |
|  | Unvaccinated | 2,257 | 1,048 | 31.9 (29.7-34.2) | Ref. 1.00 | Ref. 1.00 |

^a^Atypical squamous cells of undetermined significance or low-grade squamous intraepithelial lesion and missing/other

^b^ High-grade squamous intraepithelial lesion, atypical glandular cells, atypical squamous cells cannot exclude HSIL, adenocarcinoma in situ, or carcinoma.

^c^ Reported as ‘<n’ to prevent back-calculation of subgroups <5 patients, in line with data protection regulations.

^d^Adjusted for age at CIN2 diagnosis, calendar year, index cytology result (high-grade vs. non high-grade), income level (lowest, middle, highest) and educational level (low, intermediate, high).
